# The Factors Related to CD4+ T-Cell Recovery and Viral Suppression in Patients Who Have Low CD4+ T Cell Counts at the Initiation of HAART: A Retrospective Study of the National HIV Treatment Sub-Database of Zhejiang Province, China, 2014

**DOI:** 10.1371/journal.pone.0148915

**Published:** 2016-02-22

**Authors:** Lin He, Xiaohong Pan, Zhihui Dou, Peng Huang, Xin Zhou, Zhihang Peng, Jinlei Zheng, Jiafeng Zhang, Jiezhe Yang, Yun Xu, Jun Jiang, Lin Chen, Jianmin Jiang, Ning Wang

**Affiliations:** 1 Zhejiang Provincial Center for Disease Control and Prevention, Hangzhou, Zhejiang, China; 2 National Center for AIDS/STD Control and Prevention, Chinese Center for Disease Control and Prevention, Beijing, China; 3 Department of Epidemiology & Biostatics, School of Public Health, Nanjing Medical University, Nanjing, Jiangsu, China; Fudan University, CHINA

## Abstract

**Background:**

Since China has a unique system of delivering HIV care that includes all patients’ records. The factors related to CD4+ T-cell recovery and viral suppression in patients who have low CD4+ T cell counts at the initiation of HAART are understudied in the China despite subsequent virological suppression (viral load < 50 copies/mL) is unknown.

**Methods:**

The authors conducted a retrospective cohort study using data from the national HIV treatment sub-database of Zhejiang province to identify records of HIV+ patients. Patient records were included if they were ≥ 16 years of age, had an initial CD4 count < 100 cells/μL, were on continuous HAART for at least one year by the end of December 31, 2014; and achieved and maintained continued maximum virological suppression (MVS) (< 50 copies/ml) by 9 months after starting HAART. The primary endpoint for analysis was time to first CD4+ T cell count recovery (≥ 200, 350, 500 cells/μL). Cox proportional hazard regression was used to identify the risk factors for CD4+ T cell count recovery to key thresholds (200–350, 350–500, ≥ 500 cells/μL) by the time of last clinical follow-up (whichever occurred first), key thresholds (follow-up date for analysis), with patients still unable to reach the endpoints being censored by the end December 31, 2014 (follow-up date for analysis).

**Results:**

Of the 918 patients who were included in the study, and the median CD4+ T cell count was 39 cells/μL at the baseline. At the end of follow-up, 727 (79.2%), 363 (39.5%) and 149 (16.2%) patients had return to ≥ 200, 350, and 500 cells/μL, respectively. Kaplan-Meier analysis demonstrated that the rate of patients with CD4+ count recovery to ≥ 200, 350, and 500 cells/μL after 1 year on HAART was 43.6, 8.6, and 2.5%, respectively, after 3 years on treatment was 90.8, 46.3, and 17.9%, respectively, and after 5 years on HAART was 97.1, 72.2, and 36.4%, respectively. The median time to return to 200–350, 350–500, ≥ 500cells/μL was 1.11, 3.33 and 6.91 years, respectively. Factors of age (aHR = 0.77, 95%CI 0.61–0.97), baseline CD4+ count (aHR = 1.60, 95%CI 1.37–1.86), initial regimens, changes in regimen (aHR = 0.58, 95%CI 0.49–0.69), and inclusion of a cotrimoxazole prophylaxis (aHR = 0.66, 95%CI 0.51–0.85) were associated with CD4+ T cell count recovery.

**Conclusion:**

The proportion of patients with initially low CD4 counts after nine months of treatment and that achieved continuous virological suppression was greater than 70% for persons with CD4+ count ≥ 350. Conversely, only 35% of patients recovered to levels of 500 cells/μL after 5 years of treatment, and levels continued to rise significantly with further long-term HAART. Early HAART intervention will be necessary for achieving effective CD4+ T cell responses and optimal immunological function in HIV+ patients.

## Introduction

Highly active antiretroviral therapy (HAART) has significantly reduced mortality rates in HIV-infected patients due to virological suppression and CD4+ T cell count recovery [1]. Reducing the HIV virus load (VL) to the undetectable levels is the main goal of HAART, according to the current WHO guidelines [2]. CD4+ T cell count is a major indicator of HIV infection disease progression [3]. Patients who receive a late diagnosis have significantly poorer responses to HAART and worse prognoses [4]. However, some patients do not achieve complete CD4 recovery even with long-term virological suppression after HAART [5]. Previous studies showed that factors including age, specific drug regimen, and initial CD4 count were associated with CD4 count recovery among patients with virological suppression [6,7,8]. A cohort study indicated that a low CD4 count before treatment was a risk factor for not achieving a CD4 > 200 cells/μL [5]. Few studies have examined CD4 recovery in the context of viral suppression for more than five years. The EuroSIDA study demonstrated that patients with lower CD4 count (< 200 cells/μL) had significant rise in CD4 count even after five years of viral suppression with HAART [9]. Patients with CD4+ T counts below 100 cells/μL at initiation of HAART had over a 90% chance of recovery to above 200 cells/μL after 3 years of HAART despite VL suppression. However, only 25% of patients recovered to 500 cells/μL [10]. According to long-term HAART studies, among patients with virological suppression, only those with baseline CD4 count > 350 cells/μL returned to the normal CD4 count after six years of treatment. Conversely, patients with a lower CD4 baseline count had incomplete recovery [11].

It is still not clear whether HIV patients with severely impaired immune function (CD4+ < 100 cells/μL) can return to any significant key thresholds (≥ 200, 350, 500 cells/μL) despite achieving a long-term continued MVL suppression (VL < 50 copies/mL) by of 9 months after starting HAART. The aim of this study was to describe CD4 count changes during HAART, and its associated factors affecting the CD4 count in complete recovery to the endpoints (≥ 200, 350, 500 cells/μL) while achieving and maintaining continuous MVL suppression (VL < 50 copies/mL) in patients with lower initial CD4+ baseline count prior to HAART in Zhejiang province, China.

In 2014, 103,501 people were diagnosed with HIV-infection in China [12], with forty percent of patients have presented with a late diagnosis of AIDs (CD4 count < 200 cells/μL) in China [13]. The proportion of late diagnoses is about 30% in Zhejiang province, China. Nearly half of patients have CD4+ counts less than 100 cells/μL. Each year had a greater number of newly diagnosed patients received HAART that were severely immunocompromised Given that many patients who start HAART are severely immunocompromised, it is vital to study CD4+ T count recovery in order to modify the drug regimen or to implement more intensive follow-up as necessary. Our study focused on CD4+ T cell count recovery of patients who had initial CD4+ levels of < 100 cells/μL.

## Materials and Methods

### Database

Patients’ data were obtained from the national treatment sub-database of Zhejiang province. These included all patients who met the national treatment guidelines (CD4 count < 200 cells/μL before 2010, CD4 < 350 cells/μL from 2010 to 2014, and CD4 < 500 cells/μL in 2014) and were provided free drug treatment [14]. Patients received regular follow-up care after starting HAART. The local Centers for Disease Control or other health-care providers recorded details of treatment at baseline, at follow-up visits 0.5, 1, 2 and 3 months later and once every 3 months thereafter. The follow-up mainly included clinical evaluation and laboratory monitoring, such as CD4 testing, blood routine, liver function, and kidney function analysis. Free regular CD4 monitoring was provided every three months in the first year of treatment, and after that twice a year (the CD4 test conducted between 4 to 8 months after first year). VL testing was conducted once a year, the first VL conducted not less than 9 months after HAART, the interval between VL testing not less than 6 months after first VL testing. The local CDC or others health care organizations would complete a questionnaire after each follow-up. The above treatment database and follow-up have been described elsewhere [1,15].

### Inclusion criteria

All patients who were ≥ 16 years before treatment, had an initial CD4 count < 100 cells/μL, and had received continuous treatment for at least one year by the end of December 31, 2014 were included in the study. Patient records were also included if they achieved and maintained continuous suppression by 9 months after starting HAART.

### Exclusion criteria

Exclusion criteria were as follows: VL ≥ 50 copies/mL, if the VL detection had been interrupted (the VL testing was not conducted annually) during the treatment, and if there was loss of follow-up such as death or discontinuation of treatment. Two sensitive PCR techniques for measuring VL with the lower limit of detection of 50 copies/mL were used in Zhejiang province. Therefore, VL < 50 copies/ml was used as the maximum virological suppression in this study.

### HAART Treatment

Seven drugs divided into three categories were provided in the National Free Antiretroviral treatment Programme. The initial HAART regimens included 2 nucleoside reverse-transcriptase inhibitors (NRTIs) and 1 non-nucleoside reverse-transcriptase inhibitor (NNRTI) or 1 protease inhibitor (PIs). The NRTIs consisted of Stavudine (D4T), Zidovudine (AZT), and Tenofovir (TDF), with Lamivudine (3TC), the NNRTIs included Nevirapine (NVP) and Efavirenz (EFV), the PIs was Lopinavir/r (LPV/r). TDF and LPV/r was as the secondary line drugs before 2013. In 2013, TDF could be used as the first line treatment drug according to the National Treatment Guidelines. Meanwhile owing to the side effect of treatment or drug toxicity, D4T is no longer used in China, and patients who took D4T changed to use AZT or TDF in 2013.

### Statistical analysis

Descriptive statistics were calculated with the Student’s *t* test, One-Way ANOVA and the chi-square (χ2) test. Semi-annual changes in CD4 counts for five years after HAART to the end of December 31, 2014 were analyzed. The calculation process of CD4 count was that, for example, calculated the CD4 count in 6 months after treatment, the CD4 tested date between 4.5–7.5 months was collected, if the patient had more than one time of CD4 results in the period, the date of CD4 results closer to the 6 months was collected. We determined the mean CD4 (95% confidence interval, 95%CI) and median (interquartile, IQR) in the absolute CD4 count at baseline and semiannually thereafter.

The endpoint of the study was when CD4+ T counts first achieved key thresholds (≥ 200, 350, 500 cells/μL) and were defined complete and successful CD4 recovery. Person-years were calculated from the date of starting HAART to the time of last follow-up, whichever occurred first (≥ 200, 350, 500) key thresholds (follow-up date for analysis), with patients still unable to recovery the endpoints censored by the end December 31, 2014 (follow-up date for analysis). Kaplan-Meier plots were used to estimate the rates of CD4+ recovery to the thresholds. Cox proportional hazards regression were used to identify the factors associated with endpoints of CD4+ count recovery for participants in this study.

Univariate factors with *P* < 0.1 and factors previously shown to be associated with the CD4+ T cell count recovery after assessing collinearity and possible interactions were placed in full multivariable regression models, including age, gender, transmission route, presence of infection with *Mycobacterium tuberculosis* (TB) before treatment, initial CD4 count, WHO clinical stage, presence of change in regimen, and cotrimoxazole prophylaxis (TMP-SMZ) during the treatment and the initial regimen. Complete follow-up data were collected, which included every CD4 test results, HIV viral load measurements, and any changes in drug regimen.

All analyses were performed using STATA 12.1 (StataCorp LP, College Station, TX, USA).

### Ethical Approval

This study was reviewed and approved by the Institutional Review Board of the National Center for AIDS/STD Control and Prevention, China CDC (IRB approval number: X120331209). All the data was obtained from the national HIV treatment sub-database of Zhejiang province. All the patients who received the national free treatment at baseline, the local CDC or health-care providers would inform patient that all the treatment information may be used for the monitoring analysis. All the patients signed the informed consent.

## Results

**Fig 1:**
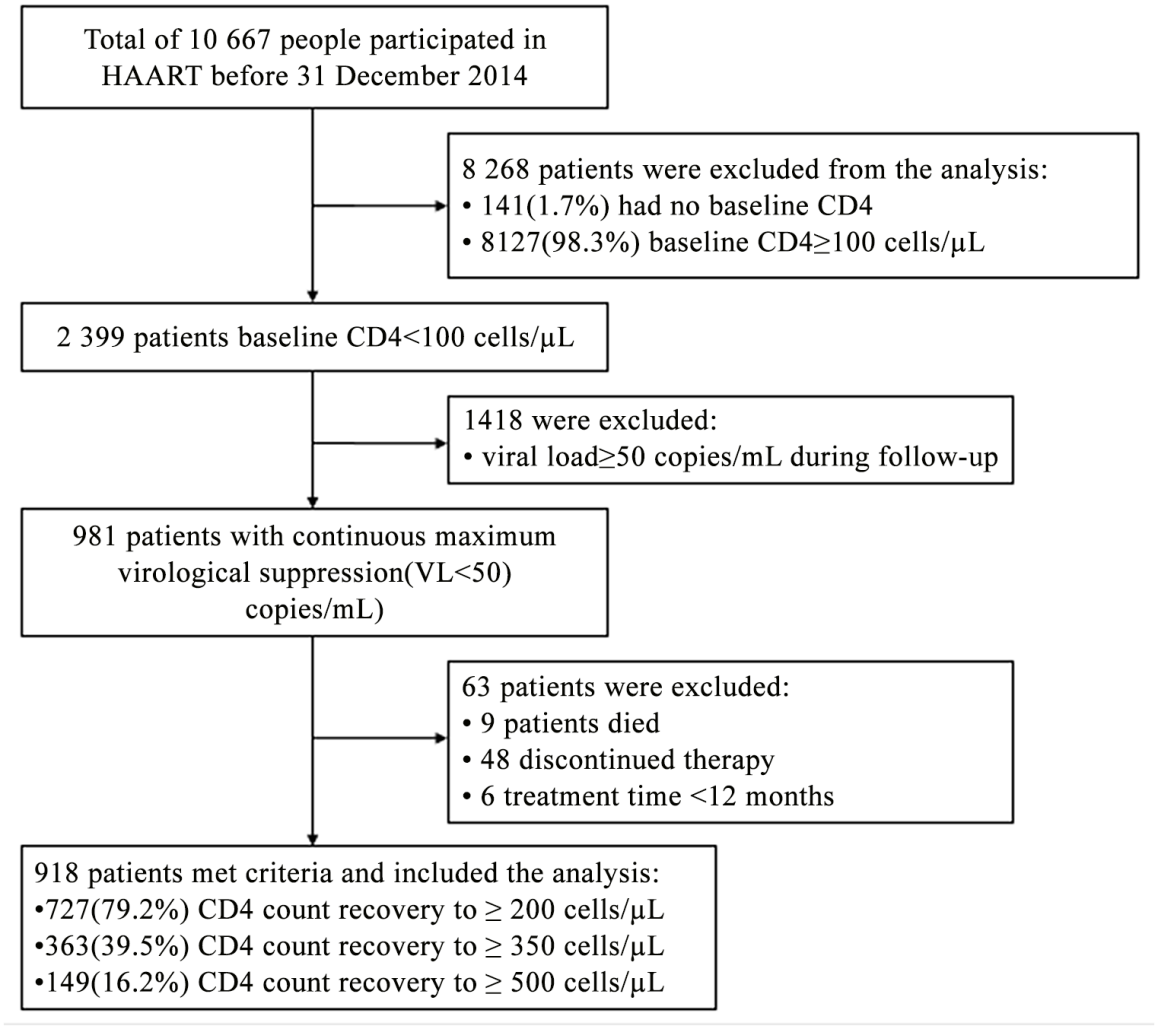
Study profile of patients.

A total of 918 patients were included in the study, and the total follow-up was 2722.1 person-years by the deadline of Dec 31, 2014, with an average 2.97 ± 1.74 years. The median treatment time was 2.5 years (IQR 1.6–4.0 years), of which the shortest was 1.0 year and the longest was 9.5 years. The person-years of CD4+ count achieved the thresholds (200–350, 350–500, ≥ 500 cells/μL) were 0.99 years (IQR 0.50–1.57 years), 1.79 years (IQR 1.16–2.74 years), and 2.19 years (IQR 1.31–3.34 years). Fig 1 shows the study profile, while Table 1 depicts the demographic characteristics of patient records by the CD4+ T cell count recovery to the endpoints (≥ 200, 350, 500 cells/μL). The median age was 39 (IQR 31–47) years. The majority of the patients were male (82.4%), the major transmission route was heterosexual transmission (64.2%).Additionally, 6.6% patients had been infected with TB one year before starting HAART. The median CD4 count was 39 cells/μL (IQR 17–68 cells/μL) at initiation; over half (60.0%) of patients had CD4+ counts less than 50 cells/μL. Nearly half (43.1%) of the subjects were at the WHO clinical stage 1 before starting treatment. The initial treatment regimens were D4T/3TC/NVP (19.7%), D4T/3TC/EFV (19.5%), AZT/3TC/NVP (26.0%), and AZT/3TC/EFV (26.6%).During the follow-up period, 31.5%, 44.4%, and 51.1% of patients changed their initial treatment regimen and achieved the endpoints of ≥ 200, 350, and 500 cells/μL, respectively. Approximately 10.9%, 15.8%, and 16.1% of the patients had been on SMZ-TMP treatment for the respective thresholds.

**Table 1:** Social demographic and characteristics of 918 patients, by the CD4+ T cell recovery to the endpoints (≥ 200, 350, 500 cells/μL).

Characteristic	All patients	No. (%)^a^ of patients(n = 918)		
		CD4+ count return to ≥ 200 cells/μL	CD4+ count return to ≥ 350 cells/μL	CD4+ count return to ≥ 500 cells/μL
**Duration of follow-up in years, median (IQR)**		0.99(0.50–1.57)	1.79(1.16–2.74)	2.19(1.31–3.34)
**Number of scheduled follow-up visits, median (IQR)**		8(6–12)	13(9–18)	1 (11–22)
**Age, median (IQR) years**	39(31–47)			
**Female**	162(17.6)			
**Transmission route**				
Heterosexual	589(64.2)			
Homosexual	284 (30.9)			
Others	45 (4.9)			
**Whether infected Tuberculosis in the prior year**				
No	857 (93.4)			
Yes	61 (6.6)			
**Baseline CD4 count (cells/μL) [median (IQR)]**	39(17–68)			
< 50	551 (60.0)			
50–99	367 (40.0)			
**WHO clinical stage**				
1	396 (43.1)			
2	149 (16.2)			
3	241 (26.3)			
4	132 (14.4)			
**Initial regimens**				
D4T+3TC+NVP	181 (19.7)			
D4T+3TC+EFV	179 (19.5)			
AZT+3TC+NVP	239 (26.0)			
AZT+3TC+EFV	244 (26.6)			
Others	75 (8.2)			
**Changes in regimen**				
No		629(68.5)	510(55.6)	449(48.9)
Yes		289(31.5)	408(44.4)	469(51.1)
**Whether subjects took TMP-SMZ During the treatment**				
No		818(89.1)	773(84.2)	770(83.9)
Yes		100(10.9)	145(15.8)	148(16.1)

WHO: World Health Organization.

^a^ All values in the table represent absolute numbers and percentages unless otherwise stated.

### CD4+ T cell count recovery

At the end of follow-up, 727 patients (79.2%) had returned to ≥ 200 cells/μL, 363 of patients (39.5%) had achieved greater than 350 cells/μL, and 149 patients (16.2%) returned to ≥ 500 cells/μL. As a result, the total rate of CD4+ count recovery the thresholds (≥ 200, 350, 500 cells/μL) were 83.7/100 person-years, 47.8/100 person-years, and 39.3/100 person-years, respectively.

**Fig 2:**
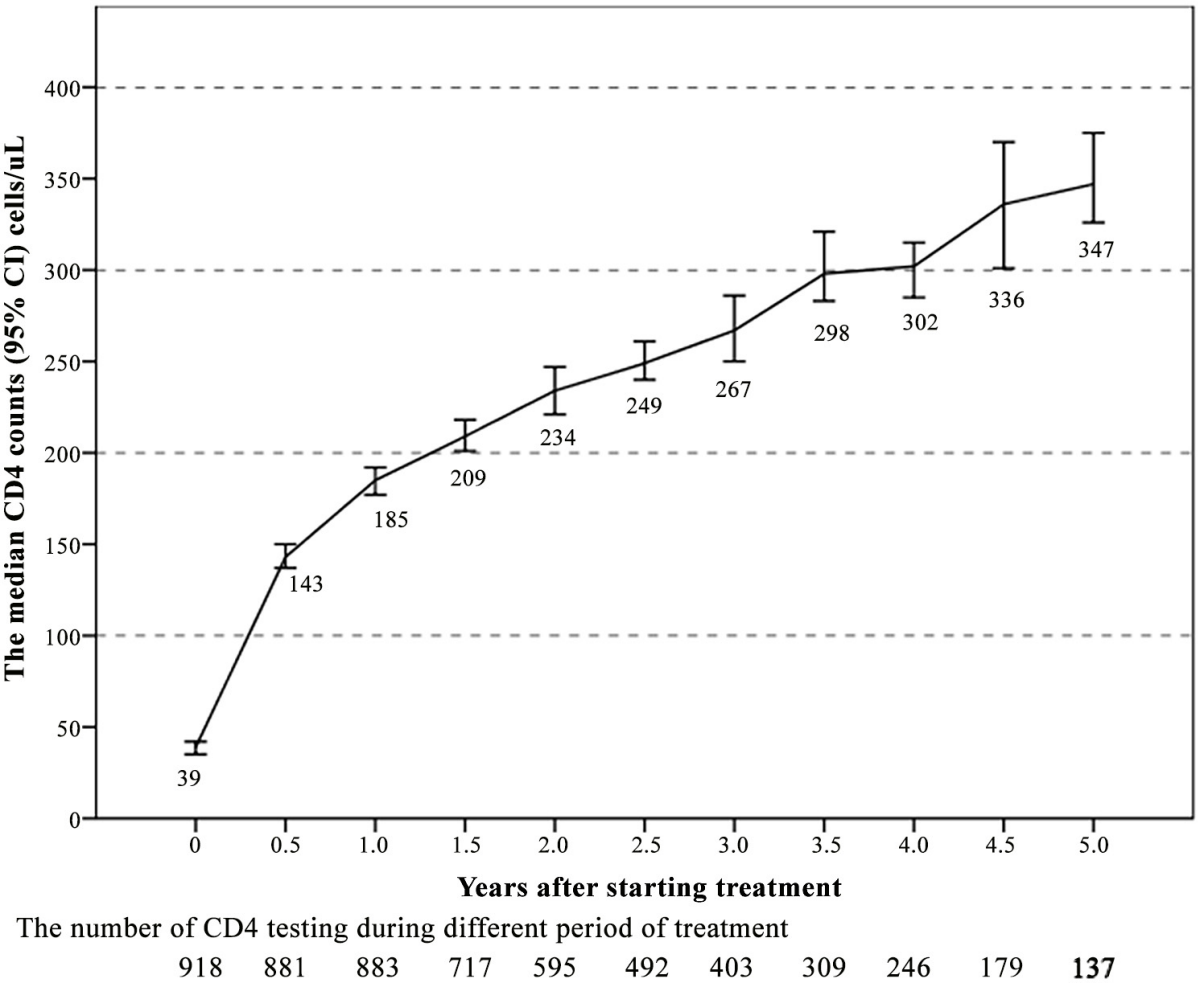
The median CD4^+^ count increased after starting treatment as observed in the 5 years of follow-up.

Fig 2 shows the CD4+ count increased during the treatment from starting HAART to 5 years on treatment. Along with HAART duration, CD4+ count continually increased, even long term after HAART initiation. The greatest CD4+ count rapid rise was seen in the first year after receiving treatment, the median CD4+ count was 39 cells/μL at baseline, up to 143 cells/μL at 0.5 year, 185 cells/μL at 1.0 year. The median CD4+ count increased slowly after that, reaching 262 cells/μL at year 2, 299 cells/μL at year 3, 331 cells/μL at year 4, and 361 cells/μL at year 5.

**Fig 3:**
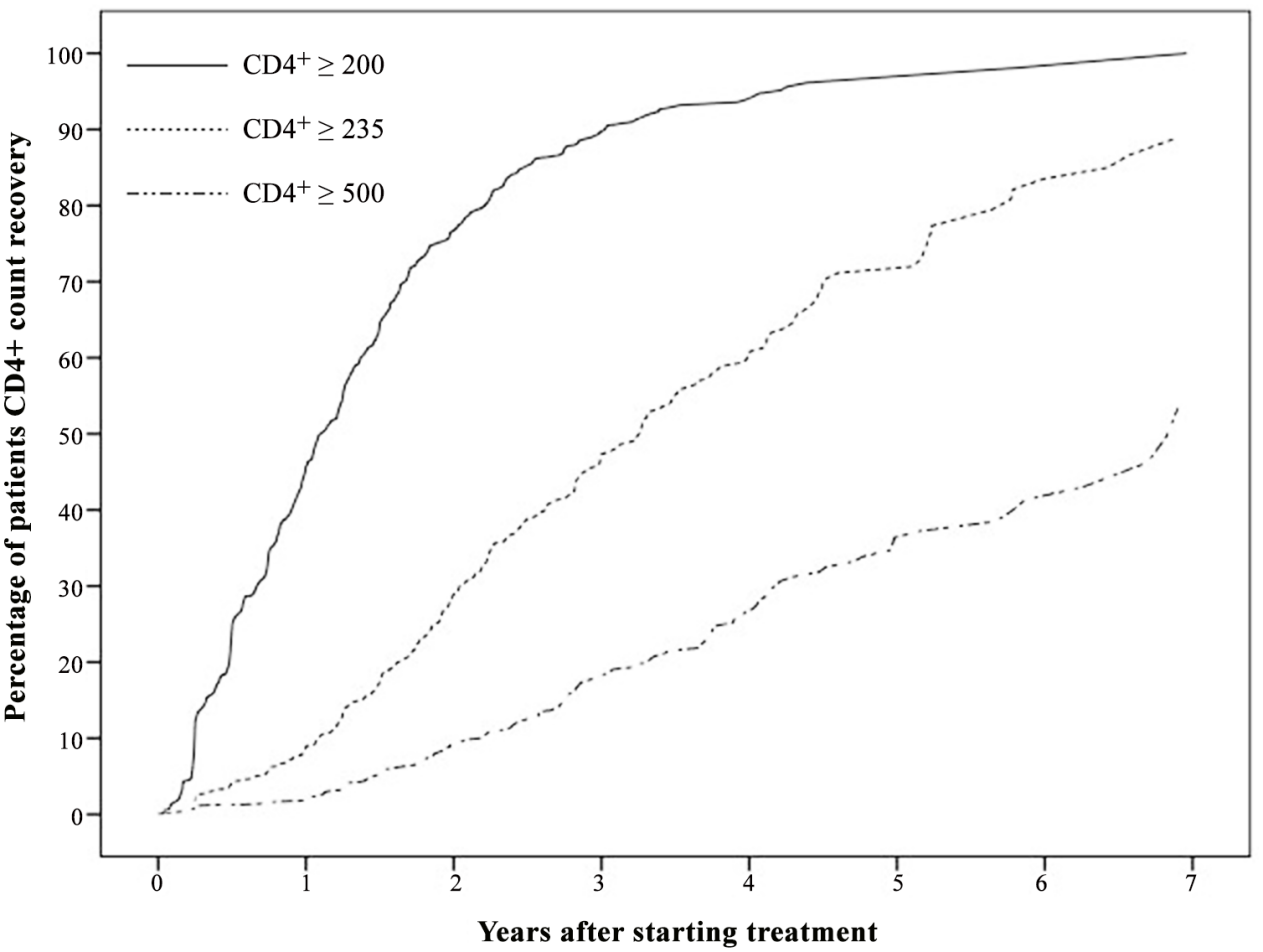
Kaplan-Meier plot of the CD4+ count recovery to the endpoints (≥ 200, 350, 500 cells/μL) from the time since starting treatment.

Fig 3 shows the Kaplan-Meier plots of the CD4+ count recovery to the endpoints (≥ 200, 350, 500 cells/μL) from the time starting treatment. Kaplan-Meier estimates that the rate of patients CD4+ count recovery to less than 200–350, 350–500, ≥ 500 cells/μL after 1 year on HAART was 43.6%, 8.6%, 2.5%, respectively, the proportion that returned to these endpoints after 3 years of treatment was 90.8%, 46.3%, 17.9%, respectively, and the proportion that recovered to these thresholds after 5 years on HAART was 97.1%, 72.2%, 36.4%, respectively. The plots indicates that the median time to return to ≥ 200, 350, 500 cells/μL was 1.11, 3.33 and 6.91 years, respectively.

Univariate Cox regression models analysis found that the factors of greater age and baseline CD4+ count (hazard ratio, HR: 1.60) were associated with the CD4+ count recovery to ≥ 200 cells/μL. Among patients who used first line drugs as the initial treatment regimen, the use of the AZT compared to D4T, of EFV compared to NVP, of changing regimen, (HR: 0.58, Table 2) and of taking TMP-SMZ during treatment (HR: 0.61) were positively associated with CD4+ count recovery to ≥ 200 cells/μL. Multivariate Cox regression analysis showed that these associations remained significant after adjusting for all potential confounders.

**Table 2:** Rate of CD4+ T cell count recovery to the endpoints (≥ 200, 350, 500 cells/μL) and its associated factors.

Factor	CD4+ count recovery to ≥ 200 cells/μL			CD4+ count recovery to ≥ 350 cells/μL			CD4+ count recovery to ≥ 500 cells/μL		
	Rate % n/N	Unadjusted HR (95% CI)	Adjusted HR (95% CI)	Rate % n/N	Unadjusted HR (95% CI)	Adjusted HR (95% CI)	Rate % n/N	Unadjusted HR (95% CI)	Adjusted HR (95% CI)
**Age, years**									
< 30	85.5(171/200)	1.0	1.0	46.5 (93/200)	1.0	1.0	19.5(39/200)	1.0	
30–	83.6(240/287)	0.90(0.74–1.10)	0.87(0.72–1.06)	46.0(132/287)	0.86(0.66–1.12)	0.84(0.64/1.10)	20.9(60/287)	0.94(0.63–1.40)	
40–	74.4(183/246)	0.71(0.58–0.88)	0.72(0.58–0.88)	33.3(82/246)	0.64(0.48–0.86)	0.62(0.46/0.84)	13.0(32/246)	0.68(0.43–1.09)	
50–	71.9(133/185)	0.70(0.56–0.88)	0.77(0.61–0.97)	30.3(56/185)	0.58(0.41–0.81)	0.71(0.51/0.99)	9.7(18/185)	0.52(0.30–0.91)	
**Baseline CD4 count (cells/μL)**									
< 50	75.9(418/551)	1.0	11.0	38.5(212/551)	1.0		16.7(92/551)	1.0	
50–99	84.2(309/367)	1.63(1.41–1.89)	1.60(1.37–1.86)	41.1(151/367)	1.20(0.97–1.48)		15.5(57/367)	1.02(0.73–1.41)	
**Initial regimens**									
D4T+3TC+NVP	95.0(172/181)	1.0	1.0	61.9(112/181)	1.0	1.0	27.1(49/181)	1.0	1.0
D4T+3TC+EFV	79.9(143/179)	0.82(0.65–1.02)	1.01(0.81–1.27)	44.7(80/179)	0.90(0.68–1.20)	1.01(0.75–1.35)	19.0(34/179)	1.04(0.67/1.62)	1.04(0.67–1.62)
AZT+3TC+NVP	78.2(187/239)	0.81(0.66–0.99)	0.78(0.63–0.96)	31.4(75/239)	0.63(0.47–0.84)	0.41(0.31–0.56)	10.9(26/239)	0.64(0.40/1.04)	0.26(0.16–0.42)
AZT+3TC+EFV	70.9(173/244)	0.77(0.62–0.95)	0.76(0.61–0.94)	33.2(81/244)	0.87(0.65–1.17)	0.59(0.44–0.80)	11.9(29/244)	0.91(0.57/1.46)	0.34(0.21–0.55)
Others	69.3(52/75)	1.03(0.75–1.40)	1.07(0.78–1.47)	20.0(15/75)	0.89(0.52–1.53)	0.57(0.33–1.00)	14.7(11/75)	1.92(0.99/3.72)	0.80(0.41–1.58)
**Changes in regimen**									
No	84.7(533/629)	1.0	1.0	47.8(244/510)	1.0	1.0	24.3(109/449)	1.0	1.0
Yes	67.1(194/289)	0.57(0.48–0.67)	0.58(0.49–0.69)	29.2(119/408)	0.44(0.35–0.54)	0.33(0.26–0.42)	8.5(40/469)	0.25(0.17–0.35)	0.14(0.09–0.21)
**Whether took TMP–SMZ During the treatment**									
No	80.6(659/818)	1.0	1.0	41.7(322/773)	1.0	1.0	17.7(136/770)	1.0	
Yes	68.0(68/100)	0.61(0.48–0.79)	0.66(0.51–0.85)	28.3(41/145)	0.66(0.48–0.92)	0.68(0.49–0.94)	8.8(13/148)	0.53(0.30–0.94)	

### The factors associated with the CD4+ T cell recovery

Univariate analysis found that the factors of CD4+ count recovery to ≥ 350cells/μL consistent with the factor of CD4+ count return to ≥ 350, cells/μL, except for the factor of baseline CD4+ count. These associated factors remained significant in the multivariate analysis.

Factors correlated with CD4+ counts returning to ≥ 500 cells/μL were analyzed by univariate Cox regression models. The initial regimens containing AZT compared to D4T, and EFV compared to NVP were positively related to CD4+ count recovery. Among patients who switched regimens, our analysis demonstrated that they were more likely to reach a CD4 count of ≥ 500. These correlations remained significant after adjustment in the multivariate analysis.

## Discussion

This is the first report that examines HIV patients CD4+ T cell count recovery to significant thresholds (≥ 200, 350, 500 cells/μL) in China with lower CD4 counts at the start of the initial HAART treatment despite achieving continuous MVL suppression. Records from the national database showed that 727 (79.2%), 363 (39.5%) and 149 (16.2%) of patients had recovery to defined endpoints (≥ 200, 350, 500 cells/μL). The total proportion of CD4+ count recovery to the thresholds were 83.7/100 person-years, 47.8/100 person-years, and 39.3/100 person-years, respectively. The CD4+ count of the majority of patients was significantly increased after treatment, even at 5 years. Specifically, rapid increase was seen in the first year of treatment, while the increases in 2–5 years were gradual. Previous studies revealed that HIV+ patients with high CD4+ initial count (≥ 350 cells/μL) could have complete recovery to either HIV-negative or normal levels. However, the corresponding recovery would be lower in patients with initial CD4 count < 200 cells/μL [16,17]. Some studies showed that CD4+ count increased quickly in the first year after treatment, but CD4+ count were not increased significantly in the second or third year [18]. The above studies in CD4+ recovery were not concerned with continuous MVL. According to the EuroSIDA data, in patients starting HAART with lower CD4+ counts (< 200 cells/μL), CD4+ count significantly rose even after 5 years of continuous VL with HAART [9]. Similar to previous studies, the authors found that patients with lower CD4 levels (<100 cells/μL) before treatment, CD4 count still increased after long-term treatment, even at five years. At present, an increase in 150 cells/μL during the first year of HAART is identified as clinical success among patients with CD4+ count more than 100 cells/μL before initial treatment [19], and for the low CD4+ count (< 100 cells/μL) before initial treatment, the CD4+ count increase is slow. A study in Netherlands showed that CD4+ recovery was worse among patients with CD4+ count below 200 cells/μL [20], but such trials were not restricted under continuous MVL suppression. This study showed that the median CD4+ count rose approximately 146 cells/μL under the continuous MVL suppression in the first year among the patients with CD4+ count less than 100 cells/μL before starting HAART.

A study from the UK (CHIC) showed that patients with CD4+ T cell count below 100 cells/μL at initial treatment and had maintained continuous VL suppression after treatment, had over 50, 14, and 3% chance of returning to ≥ 200, 350, 500 cells/μL after 1 year of treatment, respectively, and over 90, 59, and 25% after three years of treatment, respectively [10]. Our study, similar to the CHIC study, estimated that the rate of patients CD4+ count recovery to the endpoints (≥ 200, 350, 500 cells/μL) in the first year of HAART was 43.6, 8.6, and 2.5%, respectively, the proportion returning to these endpoints in the third year of treatment was 90.8, 46.3, and 17.9%, respectively. The proportion of patients that recovered to these thresholds in the fifth year of HAART was 97.1, 72.2, and 36.4%, respectively. Our findings suggest that immunological function was constantly improving among patients even at several years after HAART, and that the CD4+ count increased significantly among the patients with the long-term treatment. The study results suggest that patients with severely impaired immune function before treatment should remain in long term HAART.

We found that factors including increasing age, baseline CD4+ count, initial regimens, changes in regimen and whether TMP-SMZ was taken during the treatment were associated with CD4+ T cell count recovery. Previous studies demonstrated that increasing age is a well-recognized protective factor for CD4+ count recovery [7,21,22]. CD4+ count is more difficult to recover among older patients [20,23,24]. A study from Uganda illustrated that the CD4+ count response reduced 1.5 cells/μL per year as patients aged [25]. Data from a study of 4,041 patients showed among patients with HIV-RNA load below 50 copies/ml, the CD4+ count reduced 8.7 cells/μL as patients aged 10 years [6]. Consistent with previous studies, we found that age had an effect in lowering the rate of change in CD4+ count and resulted in incomplete recovery to ≥ 200, 350 cells/μL. This evidence might support the United States Department of Health and Human Services guidelines in 2012 that recommended HAART for all patients over 50 years old, irrespective of CD4+ count. Our results suggested that all elderly patients should receive early HAART, in order to rebuild the better immunological function, regardless of CD4+ count.

Similar to another other study [7,26], our study found that lower CD4+ count at initial treatment was a risk factor for CD4+ count recovery to ≥ 200 cells/μL, but not associated with CD4+ count recovery to ≥ 350 or 500 cells/μL. It was suggested that in patients with initial CD4+ count below 100 cells/μL, the CD4 count increased significantly in the first year, the higher initiation CD4 count level maybe an issue for CD4 recovery to ≥ 200 cells/μL. But for the CD4 count above 350 or 500 cells/μL, patients need long-term (the median years was 3.33 and 6.91) to recovery, baseline CD4+ level was no longer a factor.

Patients who used EFV for initial regimens had a higher rate of incomplete CD4+ recovery compared to the NVP regimen, while the AZT regimen compared to D4T regimen significantly affected CD4 recovery. Previous studies showed that EFV could produce higher virological suppression rates than NVP, whether the regime contained TDF or AZT [27], which might be influenced by consequence of toxicity and adherence in NVP [28]. There was no difference in the long-term effectiveness of EFV- and NVP-based HAART, however, patients initiating NVP were more likely to develop early toxicity and discontinue this drug [29]. A study which involved 2817 patients from sub-Saharan Africa demonstrated that NVP was risk factor for virological failure (aHR1.52, 95% CI 1.24–1.86) [30]. Data from a cohort study in India illustrated that use of NVP and EFV-based HAART in antiretroviral-naive Indian patients led to significant and durable rise in CD4+ cell count. Although the study was observational and non-randomized, it showed equivalent immunological response amongst NVP and EFV-based HAART [31]. A study in Uganda showed no significant difference in the CD4+ recovery between EFV and NVP after treatment [32]. Data from the HIV-CAUSAL Collaboration indicated that compared with the EFV regimen, the annual change in the CD4+ cell count (95% CI) for the NVP regimen was −11.49 cells/μL (−18.13, −4.86) [33]. In contrast, our study demonstrated that EFV was a risk factor of incomplete CD4+ recovery in patients with lower CD4+ count before treatment. Among patients with long-term virological suppression treatments, the effect of NVP was better than EFV for CD4+ count recovery, without poor compliance and toxicity.

D4T as the first line drug was used in China before 2013. However, severe side effects associated with D4T use, such as peripheral neuropathy, lactic acidosis and lipodystrophy were frequently observed and accumulated over time in several African cohorts [34,35]. A study conducted in South Africa illustrated that the mean of increasing in CD4+ count during the first year of HAART, which included D4T was 90.0 cells/μL (95% CI: 77.6, 102.3), and AZT was 69.0 cells/μL (95% CI: 62.5, 75.4) [36]. A study in Netherlands showed that patients could achieve higher (aHR = 1.30, 95% CI 1.08–1.57) CD4+ recovery with using D4T/3TC compared to AZT/3TC [8]. Our study also found similar results, AZT had higher rate of incomplete CD4+ recovery than D4T in initial regimen.

Ever changing regimen during HAART was associated with the CD4+ count recovery. Some studies suggested first antiretroviral therapy was often switched to simpler, more potent, better-tolerated regimens than the replaced regimen [37,38]. Previous studies demonstrated that side effect of patients who changed regimens during the treatment such as toxicity [39], experienced virological failure and resistance-conferring mutations within the viral genome than the patients who never changed the regimen, mainly reason due to poor compliance [40,41]. Other studies showed that the effect was significant better after changing the regimen, the median CD4+ count increased from 157 cells/μL at baseline to 307 cells/μL in 120 weeks [42]. All previous studies were not involved in the continuous HIV-RNA load MVL suppression. Our study found that patients who had changed their regimen had lower CD4+ recovery compared to those who had never changed regimens. It has been suggested the first line antiretroviral had better effects in patients with lower CD4+ count in long-term continuous virological suppression treatments.

Several studies have demonstrated that cotrimoxazole prophylaxis could protect against opportunistic infections, such as Pneumocystis *jirovecii* pneumonia, Toxoplasmosis and malaria [43]. A study from China found that cotrimoxazole prophylaxis during HAART reduced 37% mortality in HIV-infected patients [44], other studies showed the similar results [45]. Study from the DART cohort showed that patients who took co-trimoxazole did not have greater CD4+ cell count compared those who never took co-trimoxazole [46], and that co-trimoxazole had no significant effect on CD4+ recovery during long-term treatment. Our study found that patients who had used a co-trimoxazole prophylaxis had significantly lower CD4+ count recovery compared to those who had never used a cotrimoxazole prophylaxis. However, the small sample size of patients who used cotrimoxazole prophylaxis, such as patients with severely immunocompromised systems, should be taken into consideration.

Several points should be considered. The CD4+ count recovery to the key endpoints (≥ 200, 350, 500 cells/μL) was analyzed while patients achieved MVL suppression (VL < 50 copies/ml) during the treatment, thus increasing the validity of the study. The risk factor analysis of CD4+ count recovery focused on the key thresholds. However, this study has several limitations. We identified all patients with initial CD4+ T count < 100 cells/μL in the analysis, but over half of the patients did not achieve continuous MVL suppression or did not meet other criteria, were excluded. This means that our findings focus on patients with lower CD4+ T cell count in the initial HAART, while achieving continuous MVL suppression by the time of 9 months after treatment. Owing to the side effect of treatment or drug toxicity, D4T was no longer used in China in 2013, and patients who took D4T changed to use AZT or TDF, so consideration should be given to whether TDF might affect the CD4+ count recovery. This was also an observational study therefore patients were not randomly chosen to start initial treatment regimens which were later changed during the course of treatment, as such findings should be interpreted with caution.

In conclusion, this is the first study that examines the CD4+ T cell count recovery in HIV+ patients with lower CD4+ T cell count (< 100 cells/μL) at initial treatment, achieving and maintaining continuous virological suppression by the 9 months after HAART in China. The proportion of patients with initially low CD4 counts after nine months of treatment and that had achieved continuous virological suppression was greater than 70% for persons with CD4+ count ≥ 350. Conversely, only 35% of patients recovered to 500 cells/μL after 5 years of treatment, and continued to significantly rise with long-term HAART. Early HAART intervention will be necessary for achieving effective CD4+ T cell responses and optimal immunological function in HIV+ patients.
